# Genetic association of *LOC100130476* rs80213143 with susceptibility and renal involvement in systemic lupus erythematosus

**DOI:** 10.3389/fgene.2026.1609849

**Published:** 2026-04-01

**Authors:** Xiao-Xue Zhang, Jun-Peng You, Yong-Chun Li, Hong-De Xu, Xin-Yi Chen, Kai-Le Feng, Zhi-Qiu He, Zhan-Zheng Zhao, Yuan-Yuan Qi

**Affiliations:** 1 Department of Nephrology, The First Affiliated Hospital of Zhengzhou University, Zhengzhou, Henan, China; 2 Zhengzhou University, Zhengzhou, Henan, China; 3 Laboratory of Nephrology, The First Affiliated Hospital of Zhengzhou University, Zhengzhou, Henan, China; 4 Ministry of Education of China, Institute of Drug Discovery and Development, Zhengzhou University, Zhengzhou, Henan, China; 5 School of Pharmaceutical Sciences, Zhengzhou University, Zhengzhou, Henan, China

**Keywords:** genotyping, LOC100130476, lupus nephritis, single nucleotide polymorphisms, systemic lupus erythematosus

## Abstract

**Backgrounds:**

Systemic lupus erythematosus (SLE) is an autoimmune disease with multi-organ involvement, and lupus nephritis (LN) is a severe manifestation. Long non-coding RNAs (lncRNAs) have been implicated in regulating immune responses in autoimmune diseases. LOC100130476, a lncRNA located on chromosome 6q23.3, has been linked to inflammation and cancer progression, but its role in SLE and LN remains unclear.

**Methods:**

We studied the association between the rs80213143 variant at LOC100130476 and SLE susceptibility in a Chinese Han cohort, using SNP genotyping and Bonferroni correction for multiple comparisons. Functional annotations were conducted to explore the effects of rs80213143 on transcription factor binding and gene expression. eQTL analysis was performed to assess the variant’s impact on immune cell gene expression.

**Results:**

Within LOC100130476, the strongest association was observed at rs80213143 (p = 2.5 × 10^−7^), which was successfully replicated (p = 2.64 × 10^−9^) in an independent cohort. The combined analysis of both discovery and replication cohorts reinforced the genetic association (p_meta_ = 2.04 × 10^−14^). The risk C allele was linked to more severe renal involvement, including higher 24-h proteinuria and serum creatinine levels. Functional annotations indicated that rs80213143 potentially influences immune cell functionality through regulatory motif alterations. The expression of *LOC100130476* was abnormally upregulated in the whole blood of SLE patients, particularly in lupus nephritis patients. Moreover, the expression of *LOC100130476* was significantly upregulated in the biopsy samples of lupus nephritis patients. Differentially expressed genes in whole blood between SLE patients and healthy donors, positively associated with *LOC100130476* expression, were significantly enriched in pathways involving T cell receptor signaling, antigen presentation, interferon response, and apoptosis. Furthermore, *LOC100130476* showed positive associations with genes differentially expressed between LN patients' renal biopsy tissues and adjacent normal renal tissues, enriched in leukocyte-mediated immunity, inflammatory responses, extracellular matrix and tissue repair pathways, and the PI3K signaling network.

**Conclusion:**

The rs80213143 variant in LOC100130476 is associated with SLE susceptibility and renal involvement. Its elevated expression in lupus nephritis suggests it may be an important factor in disease pathogenesis and a potential biomarker for lupus nephritis.

## Introduction

Systemic lupus erythematosus (SLE) is a prototypical autoimmune disease characterized by the production of autoantibodies against nuclear antigens, leading to inflammation and multi-organ involvement. The pathogenesis of SLE, while not fully understood, is influenced by genetic, environmental, and immunological factors. Recent advances have highlighted the role of long non-coding RNAs (lncRNAs) in the pathogenesis of SLE. LncRNAs, crucial members of the non-coding RNA family, are RNA transcripts exceeding 200 nucleotides without protein-coding capability (Kapranov et al., 2007). Identified as immune regulators, their dysregulation plays a role in autoimmune diseases, including SLE (Tsai et al., 2020).

Previous studies have demonstrated abnormalities in lncRNA expression in SLE patients. In peripheral blood mononuclear cells (PBMCs), 8,868 lncRNAs showed differential expression between SLE patients and healthy donors, with 3,657 upregulated and 5,211 downregulated (Luo et al., 2018). Similarly, in monocyte-derived dendritic cells (moDCs), 163 lncRNAs exhibited differential expression between SLE and controls (Wang et al., 2018). The versatile role of lncRNA in immunity and inflammation is highlighted by its correlation with SLE disease activity. Specifically, the expression levels of ENST00000604411.1, ENST00000501122.2, NEAT1 in moDCs, and lnc7514 in PBMCs positively correlated with clinical disease activity (Wang et al., 2018; Zhang et al., 2016; Wang et al., 2019). In patients positive for anti-dsDNA, lnc7514 levels were markedly lower than in those negative for anti-dsDNA (Wang et al., 2019). Furthermore, lnc3643 expression levels were associated with C-reactive protein and erythrocyte sedimentation rates in SLE patients (Wang et al., 2019).

Evidence from genome-wide association studies (GWAS) and candidate gene analyses has highlighted the association of single nucleotide polymorphisms (SNPs) with susceptibility to SLE. The variant rs13259960, situated in an intronic enhancer region of SLEAR, has been linked to increased susceptibility to SLE (P = 1.03 × 10^−11^), influencing SLEAR expression that correlates positively with cell death in the peripheral blood of SLE patients (Fan et al., 2020). Similarly, the risk alleles rs205764 and rs547311, located in the promoter region of linc00513, are known to elevate linc00513 expression by enhancing promoter activity, contributing to SLE. Linc00513 acts as a positive regulator of the type I interferon pathway and correlates positively with the IFN score in SLE patients (Xue et al., 2018).

We reviewed previous GWAS data with a focus on lncRNA-coding genes (Sun et al., 2016). Among the Chinese Han population from Beijing, rs80213143 at LOC100130476 emerged as a locus significantly associated with increased susceptibility to systemic lupus erythematosus (SLE) (p = 2.5 × 10^−7^, OR 2.63, 95% CI 1.80–3.84). Furthermore, this study replicated the genetic association between rs80213143 and SLE susceptibility and explored the role of LOC100130476 in the pathogenesis and progression of SLE.

## Methods

### Participants

We utilized genetic association results from a previous GWAS (Sun et al., 2016) involving a Beijing cohort as our discovery set, which included 490 SLE patients and 493 controls. These GWAS summary-level association data were obtained from a publicly available, previously published study (Sun et al., 2016) and were used for secondary analysis in the present study. An independent replication cohort, consisting of 1003 SLE patients and 815 geographically matched, unrelated healthy controls, was recruited from Henan, Central China. All SLE participants fulfilled the 1982 American College of Rheumatology (ACR) classification criteria for SLE (Tan et al., 1982), as revised by Hochberg in 1997 (Hochberg, 1997). Ethical approval for the study was granted by the Medical Ethics Committee of Zhengzhou University First Hospital (2019-KY-247), ensuring compliance with the Declaration of Helsinki. Informed consent was obtained from all participants.

### Polymorphism selection and genotyping

In the discovery cohort, we included all SNPs within the LOC100130476 gene region covered by the ImmunoChip, as outlined previously (Sun et al., 2016). The publicly available GWAS datasets used in this study were derived from previously published analyses in which PCA-based adjustment had already been performed. In the replication cohort, the SNP rs80213143 at LOC100130476, identified as the most significant, was genotyped using the Sequenom MassARRAY system, achieving a genotyping completion rate of over 97%.

### Variant annotation and expression analysis across immune cells

We conducted annotations of the variants' regulatory elements through HaploReg v4.2 (Ward and Kellis, 2012) and RegulomeDB v2.2 databases (Boyle et al., 2012). For assessing cell type-specific expression quantitative trait loci (eQTL) and the expression of target genes, ImmuNexUT (Immune Cell Gene Expression Atlas from the University of Tokyo) (Ota et al., 2021), which encompasses data on 28 immune cell types from patients with various immune diseases and healthy individuals, was utilized.

### Gene expression analysis

Total RNA was extracted and isolated from whole blood and renal tissues (renal biopsy samples and paracancerous kidney tissues) using TRIzol Reagent (Life Technologies) following the manufacturer’s protocol. Whole genome RNA sequencing (RNA-seq) was performed with PE150 (Illumina, San Diego, CA, USA) (Qi et al., 2021). LOC100130476 has been characterized as a polyadenylated long non-coding RNA transcript (PMID: 30594489), thereby allowing for its accurate detection and quantification in poly(A)-selected RNA-seq datasets, including those utilized in our analysis. For the whole blood sequencing, the cohort comprised 99 individuals, including 57 LN patients, 18 SLE patients without renal impairment, and 24 healthy controls. In the case of renal tissue sequencing, the sample set included seven lupus nephritis renal biopsy tissues and four adjacent normal renal tissues. This cohort was previously utilized in our earlier studies (Qi et al., 2021; Singh et al., 2024), where RNA-seq data generation and processing were conducted.

### Statistical analysis

Hardy-Weinberg Equilibrium (HWE) was evaluated using a goodness-of-fit chi-square (χ2) test. The P value for HWE in the control population of rs80213143 was 0.654, indicating adherence to HWE. Statistical significance for genetic association analysis was determined using the Chi-square test. Analysis of LOC100130476 expression in whole blood and renal tissues was conducted using independent samples t-tests or one-way ANOVA, as appropriate, with a significance threshold set at p < 0.05. Data analysis was performed using SPSS software (Version 19.0; SPSS Inc., IL).

## Results

### Association between *LOC100130476* gene polymorphisms and SLE susceptibility

LOC100130476 is located on chromosome 6, positions 138,144,807 to 138,189,370, as defined by RefSeq genes. Out of 34 SNPs within *LOC100130476* spanning approximately 45 kb and covered by the ImmunoChip, 31 were successfully genotyped (Sun et al., 2016) (Supplementary Table S1). Twenty-three SNPs were found to be associated with SLE susceptibility with p-values <0.05 (Supplementary Table S1).

**FIGURE 1:**
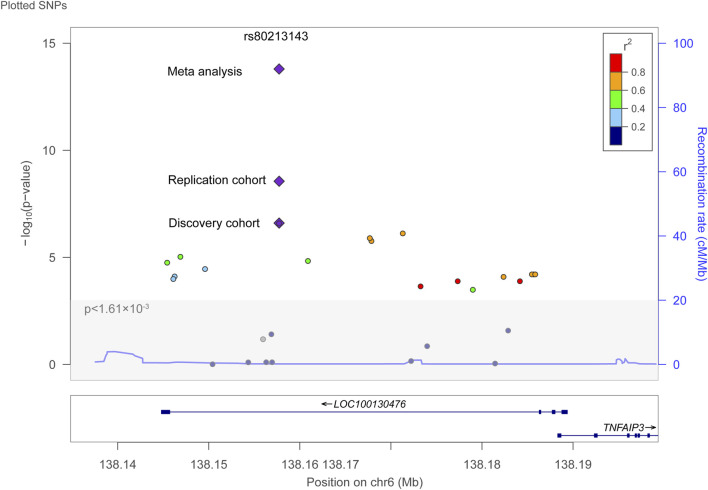
Genetic association analysis of LOC100130476 polymorphisms with SLE susceptibility. The plot presented p-values on a -log10 scale plotted against chromosomal positions (Mb) on chromosome 6, highlighting the SNPs analyzed. The SNP rs80213143 was emphasized, exhibiting the most significant association with SLE susceptibility within the LOC100130476 locus. A horizontal line marked the Bonferroni-corrected significance threshold at p < 1.61 × 10^−3^. This visualization was created using LocusZoom.

To adjust for multiple comparisons in our genetic association analysis, we applied the Bonferroni correction method to reduce Type I error, setting a more stringent significance level. Accordingly, a p-value of less than 1.61 × 10^−3^ was deemed significant after Bonferroni adjustment. Of the 31 SNPs analyzed, 20 were significantly associated with SLE susceptibility following Bonferroni correction (Figure 1).

The strongest association was detected at rs80213143 (p = 2.5 × 10^−7^, OR 2.63, 95%CI 1.80–3.84) (Sun et al., 2016), which was successfully replicated in an independent cohort from Henan, Central China (p = 2.64 × 10^−9^, OR 2.32, 95%CI 1.74–3.07). A meta-analysis combining the discovery and replication cohorts underscored this genetic association (p_meta_ = 2.04 × 10^−14^, OR 2.42, 95%CI 1.93–3.04) as shown in Figure 1 and Table 1.

**TABLE 1:** Association of *LOC100130476* polymorphisms with systemic lupus erythematosus susceptibility.

SNP	Minor allele	Discovery population (SLE vs. control, 490/493)			Replication population (SLE vs. control, 1003/815)			Meta analysis (SLE vs. control, 1493/1308)	
		MAF (%)	P	OR (95% CI)	MAF (%)	P	OR (95% CI)	P	OR (95% CI)
rs80213143	C	10/4.1	2.50 × 10^−7^	2.63 (1.80–3.84)	9.57/4.37	2.64 × 10^−9^	2.32 (1.74–3.07)	2.04 × 10^−14^	2.42 (1.93–3.04)

### Association between LOC100130476 rs80213143 genotypes and clinical phenotypes in SLE patients

Given the robust genetic association of rs80213143 with SLE, we next evaluated its relationship with clinical and laboratory phenotypes (Table 2). The results revealed that the C allele, identified as the risk allele, was significantly associated with more severe renal involvement. Specifically, patients carrying the C allele (CC + CG group) exhibited higher 24-h proteinuria levels [median (IQR): 0.69 (0.05–2.12) g vs. 0.60 (0.25–1.30) g, p = 0.014] and higher serum creatinine levels [median (IQR): 64 (48–72) µmol/L vs. 55 (47–66)µmol/L, p = 0.018] compared to the GG group.

**TABLE 2:** Association between LOC100130476 rs80213143 genotypes and clinical phenotypes in SLE patients.

Clinical phenotypes	CC + CG (n = 176)	GG (n = 796)	p value
Demographics			
Age at onset (years)	30.5 ± 11.9	31.6 ± 13.2	0.265
Male gender (%)	5.1	7.7	0.236
Mucocutaneous			
Malar rash (%)	28.4	24.0	0.220
Discoid rash (%)	0.6	0.8	0.792
Photosensitivity (%)	4.5	4.0	0.751
Oral ulcers (%)	9.1	6.8	0.284
Musculoskeletal			
Nonerosive arthritis (%)	27.3	28.1	0.816
Serositis	​	​	​
Pleuritis/Pericarditis (%)	8.0	7.9	0.819
Neurologic disorder (%)	4.5	3.3	0.403
Renal			
24 h proteinuria (g)	0.69 (0.05–2.12)	0.60 (0.25–1.30)	**0.014**
Serum creatinine (umol/L)	55 (47–66)	64 (48–72)	**0.018**
Hematologic			
Leukopenia (%)	20.6	26.3	0.118
Thrombocytopenia (%)	19.4	25.6	0.087
Immunologic			
Anti-dsDNA titre (%)	66.9	61.9	0.247
Anti-Sm antibodies (%)	17.5	17.8	0.923
C3 level (g/L)	0.70 + 0.36	0.73 + 0.36	0.360
C4 level (g/L)	0.13 + 0.13	0.14 + 0.13	0.210

Bold values indicate statistical significance (p < 0.05).

Preliminary findings further suggested that patients with the C allele tended to have earlier disease onset and higher prevalence of mucocutaneous manifestations, neurologic disorders, and serositis. Additionally, a trend toward higher anti-dsDNA positivity rates and lower serum complement levels (C3 and C4) was observed in the CC + CG group. However, these differences were not statistically significant.

### Functional annotation of SLE-associated SNPs

To explore the functional relevance of the associated SNPs, we utilized RegulomeDB for annotation of known and predicted regulatory elements. According to RegulomeDB, rs80213143 was assigned a rank of five and a score of 0.005, indicating its location in 833 chromatin state regions and five motif regions (Table 3). We extended our functional annotation to include all SNPs genotyped in the discovery cohort (Table 3). The ranking and scoring system provided different metrics to evaluate the regulatory potential of a variant. Notably, other variants such as rs653520 (rank 1b) and rs73564258 (score 0.70044) exhibited even stronger evidence of regulatory potential.

**TABLE 3:** Functional annotations of SNPs analyzed in *LOC100130476* gene with RegulomeDB.

SNPs	Rank	Score	ChIP data	Chromatin state	Accessibility	Motifs	QTL data
rs653520	1b	0.3225	106	833	883	6	4
rs657180	1f	0.19549	0	833	27	2	3
rs657597	1f	0.22271	0	833	16	0	3
rs2788288	1f	0.55436	23	833	148	0	4
rs72980748	1f	0.66703	2	833	8	1	2
rs17779870	1f	0.55324	1	833	0	0	2
rs111883038	1f	0.55436	55	833	469	0	1
rs9494883	1f	0.55436	8	833	7	0	2
rs600144	1f	0.55324	63	833	0	0	2
rs7753873	1f	0.55436	6	833	11	0	4
rs7767264	1f	0.55324	32	833	0	0	2
rs11970411	1f	0.22271	0	833	1	0	2
rs7774101	1f	0.42858	11	833	0	5	2
rs9494886	1f	0.55436	119	833	11	0	2
rs59699063	1f	0.55324	27	833	0	0	2
rs61593413	1f	0.55324	39	833	0	0	2
rs59693083	1f	0.66703	135	833	26	1	2
rs73564258	2b	0.70044	57	833	2	2	0
rs56232106	3a	0.55134	1	833	1	1	0
rs80126770	4	0.60906	56	833	519	0	0
rs9376303	4	0.60906	4	833	8	0	0
rs9376304	4	0.60906	67	833	77	0	0
rs7746779	5	0.13454	0	833	1	0	0
rs80213143	5	0.005	0	833	1	5	0
rs57163170	6	0.32023	0	833	0	10	0
rs670369	7	0.51392	0	833	0	0	3
rs9389536	7	0.18412	0	833	0	0	0
rs6918329	7	0.18412	0	833	0	0	0

**FIGURE 2:**
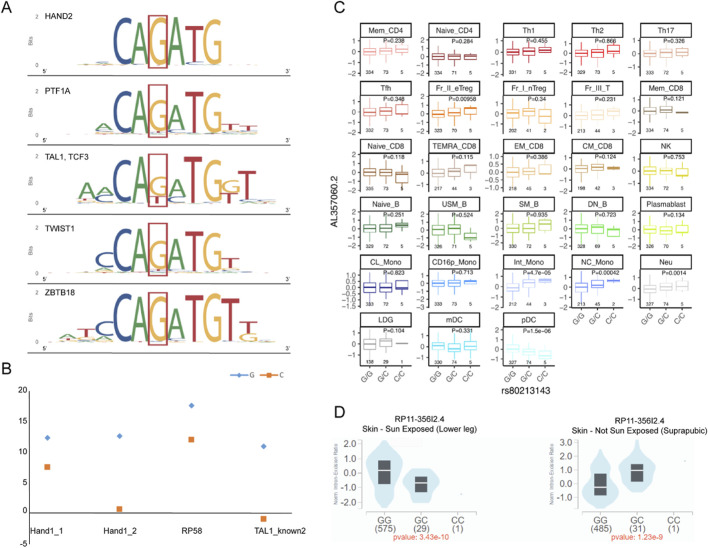
Analysis of the regulatory potential of rs80213143 and its QTL effects. **(A)** Sequence motifs for transcription factors HAND2, PTF1A, TAL1/TCF3, TWIST1, and ZBTB18 were potentially impacted by the rs80213143 locus as annotated by RegulomeDB. **(B)** Binding affinity scores for motifs Hand1_1, Hand1_2, RP58, and TAL1_known2 demonstrated reduced binding affinity when the reference G allele was substituted with the C allele. **(C)** Expression levels of LOC100130476 (also known as AL357060.2 in the Immune Cell Gene Expression Atlas) across various immune cell types showed significant differences between genotypes observed in intermediate monocytes (Int Mono), non-classical monocytes (NC Mono), and neutrophils (Neu), indicating a potential cell type-specific eQTL effect of the rs80213143 locus on LOC100130476 expression. **(D)** Violin plots represented the normalized expression ratio of the lncRNA RP11-356I2.4 transcript in skin tissue samples from individuals with varying genotypes at rs80213143. The left panel showed data from sun-exposed lower leg skin, and the right panel showed data from non-sun-exposed (suprapubic) skin.

Allelic variation at rs80213143 (G vs. C) is predicted to alter binding affinities for several transcription factors including HAND2, PTF1A, TAL1, TCF3, TWIST1, and ZBTB18 (Figure 2A). Using HaploReg, we examined the regulatory motif alterations in greater detail. The analysis revealed that substitution of the reference G allele with the risk C allele resulted in diminished binding affinities for the Hand1_1 motif (score reduced from 12.4 to 7.6) and Hand1_2 motif (12.7–0.7). Similar reductions were observed for the RP58 motif (from 17.7 to 12.1) and the TAL1_known2 motif (from 11 to −0.9) (Figure 2B). These results suggest that the risk allele may impair transcription factor binding, potentially altering downstream gene expression.

### eQTL analysis and cell type-specific effects of rs80213143

Subsequently, we focused on investigating the quantitative trait locus (QTL) effects of the rs80213143 variant. SLE is a prototypical autoimmune disease, and we subsequently investigated the impact of rs80213143 on expression across various human immune cell types, according to results from the Immune Cell Gene Expression Atlas database, developed by Ota et al. at the Department of Allergy and Rheumatology, Graduate School of Medicine, the University of Tokyo (Ota et al., 2021) (Figure 2C). Several plots show a notable difference in expression between genotypes. In “Int Mono” (intermediate monocytes), the expression level is significantly different between the genotypes, with a p-value of 4.7e-05. Similarly, “NC Mono” (non-classical monocytes) and “Neu” (neutrophils) show significant eQTL effects with p-values of 0.00042 and 0.0014, respectively, suggesting that the gene expression levels in these cell types are influenced by the genotype at rs80213143. However, most of the other cell types do not show statistically significant differences in gene expression levels across genotypes. This implies that the impact of rs80213143 on the expression of *LOC100130476* may be cell type-specific.

Our examination of the GTEx database indicated a significant eQTL effect of rs80213143 on the expression of RP11-356I2.4, a transcript variant of LOC100130476 (Figure 2D). The eQTL effect was observed to be modulated by environmental exposure, particularly to sunlight, which is known to exacerbate the manifestation of SLE. The data revealed that the risk allele C at rs80213143 was associated with lower expression levels of RP11-356I2.4 in skin exposed to sunlight (lower leg) compared to skin not exposed to sunlight (suprapubic area). This finding suggested a potential interaction between the environmental factor of sunlight exposure and the genetic risk allele in the pathogenesis of SLE, with the allele C potentially conferring a greater decrease in expression under sun-exposed conditions.

### *LOC100130476* expression in SLE and lupus nephritis

**FIGURE 3:**
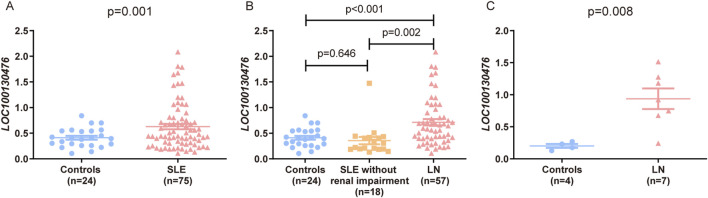
Expression analysis of LOC100130476 in SLE patients and controls, with a focus on LN. The expression of LOC100130476 was analyzed in whole blood samples from SLE patients compared to controls **(A)**, and further stratified between SLE patients without renal impairment, lupus nephritis (LN) patients, and controls **(B)**. Additionally, LOC100130476 expression was compared between renal biopsy samples from LN patients and paracancerous kidney tissues serving as controls **(C)**.

We next compared LOC100130476 expression levels in peripheral blood and renal tissues. LOC100130476 expression was significantly higher in SLE patients than in controls (p < 0.001) (Figure 3A). Notably, lupus nephritis patients exhibited an increased level of LOC100130476 compared to both controls (p < 0.001) and SLE patients without renal impairment (p = 0.002) (Figure 3B). However, no significant difference in LOC100130476 expression was observed between controls and SLE patients without renal impairment (p = 0.646) (Figure 3B). Additionally, in renal biopsy samples, LOC100130476 expression was markedly elevated in lupus nephritis patients compared to paracancerous kidney tissue used as control (p = 0.008) (Figure 3C).

### Correlation of *LOC100130476* with pathogenic pathways in SLE and LN

**FIGURE 4:**
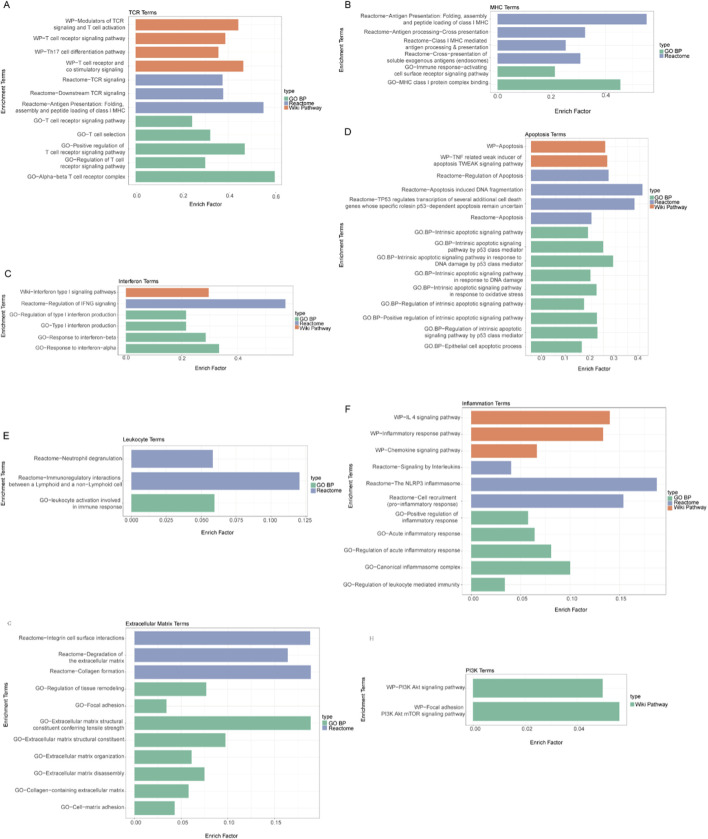
Enrichment analysis of pathways correlated with LOC100130476 expression in SLE and LN **(A)** T Cell Receptor Signaling Pathways: This panel illustrated the enrichment of genes that were positively correlated with LOC100130476 expression in pathways related to the T cell receptor (TCR) complex and signaling, including the positive regulation of TCR signaling and T cell selection. These pathways were crucial for the activation and differentiation of T cells, which played a central role in the immune response in SLE. **(B)** Antigen Presentation Pathways: The figure depicted the enrichment of pathways involved in antigen presentation, including the folding, assembly, and peptide loading of class I MHC molecules. These processes were essential for the activation of the adaptive immune response and the presentation of antigens to T cells. **(C)** Interferon Signaling Pathways: This panel showed the enrichment of genes in pathways related to the response to interferons, including both type I and type II interferons. Interferons were critical cytokines involved in the immune response to viral infections and had been implicated in the dysregulation of immune responses in SLE. **(D)** Apoptosis Pathways: The figure highlighted the enrichment of pathways associated with the apoptotic process, including the intrinsic apoptotic signaling pathway and its regulation by p53 class mediators. Apoptosis was a key process in maintaining immune homeostasis, and its dysregulation could contribute to autoimmune diseases like SLE. **(E)** Immunoregulation and Leukocyte Activation Pathways: This panel displayed the enrichment of pathways governing immunoregulation, leukocyte activation, and cell adhesion and migration. These pathways were involved in the modulation of immune responses and the recruitment of immune cells to sites of inflammation, which were critical in the pathogenesis of LN. **(F)** Inflammatory Response Pathways: The figure showed the enrichment of genes in pathways related to the inflammatory response, including the acute inflammatory response and its positive regulation. Inflammation was a hallmark of LN, and the pathways highlighted here contributed to the chronic inflammation observed in this condition. **(G)** Extracellular Matrix and Tissue Repair Pathways: This panel illustrated the enrichment of pathways involved in the extracellular matrix (ECM) and tissue repair, including ECM organization and cell-matrix adhesion. These processes were important in the structural integrity of tissues, and their dysregulation could lead to tissue fibrosis, a common complication in LN. **(H)** PI3K Signaling Pathways: The figure depicted the enrichment of pathways related to the PI3K signaling network, which played a role in various cellular processes including cell survival, proliferation, and metabolism. Dysregulation of these pathways could contribute to the pathogenesis of LN.

To further elucidate how LOC100130476 may contribute to the pathogenesis of SLE and LN, we conducted a correlation analysis between differentially expressed genes in SLE and LN and *LOC100130476* expression (Figure 4).

Upon analyzing transcriptome sequencing data for SLE compared to healthy control whole blood, we identified genes differentially expressed and positively correlated with LOC100130476 expression, significantly enriched in pathways related to cell cycle regulation, DNA damage response, transcription and translation processes, immune response, cellular signaling, and the biogenesis and maintenance of organelles. Conversely, pathways enriched by genes negatively correlated with LOC100130476 predominantly pertained to erythrocyte development and heme metabolism. Given the characteristics of SLE, we noted that the positively associated pathways include T cell receptor (Figure 4A), antigen presentation (Figure 4B), interferon (Figure 4C), and apoptosis (Figure 4D) suggesting a potential role for *LOC100130476* in promoting the progression of SLE.

In the analysis of transcriptome sequencing results for lupus nephritis renal biopsy tissues and adjacent normal renal tissues, genes that were differentially expressed and positively correlated with *LOC100130476* expression were significantly enriched in pathways governing immunoregulation, cell adhesion and migration, extracellular matrix (ECM) dynamics, signal transduction, inflammation, and phagocytosis. These included leukocyte-mediated immunity (Figure 4E), inflammatory responses (Figure 4F), pathways related to the extracellular matrix and tissue repair (Figure 4G), and the PI3K (Phosphoinositide 3-kinases) signaling network (Figure 4H). Such pathways are recognized contributors to the pathogenesis and progression of LN. Conversely, pathways negatively correlated with *LOC100130476* expression, predominantly involving metabolic and respiratory functions, appeared to be less directly implicated in SLE pathophysiology. This marked divergence in pathway characteristics suggests that *LOC100130476* might significantly influence the promotion of pathogenic mechanisms underlying LN.

## Discussion

In this study, we demonstrated a robust association between the rs80213143 variant at the LOC100130476 locus and susceptibility to SLE. Notably, the risk C allele was not only linked to an increased overall risk for SLE (p < 2.04 × 10^−14^) but was also significantly associated with more severe renal involvement. Carriers of the C allele exhibited higher levels of 24-h proteinuria and elevated serum creatinine, indicating a potential role of this variant in the pathogenesis of LN. Although trends toward earlier disease onset and increased prevalence of mucocutaneous, neurologic, and serositis manifestations were observed in C allele carriers, these associations did not reach statistical significance, warranting further investigation in larger cohorts. It should be noted that the association signal for rs80213143 in the discovery cohort did not reach the conventional genome-wide significance threshold; however, the finding was consistently replicated in an independent cohort and further strengthened by meta-analysis, providing robust genetic evidence for the involvement of this locus in SLE susceptibility.

The SNP rs80213143 is located within the *TNFAIP3* gene region on chromosome 6, a well-established risk haplotype for SLE and other immune-mediated diseases (Ray et al., 2020). Previous studies have reported associations between rs80213143 and several immune-related disorders, including rheumatoid arthritis (Ray et al., 2020), asthma (Ray et al., 2020), and systemic sclerosis (Terao et al., 2017), suggesting its potential functional role in immune dysregulation. However, rs80213143 has not been directly associated with SLE. Although other variants within the *TNFAIP3* haplotype have been linked to SLE, the specific role of rs80213143 remains underexplored. This study is the first to demonstrate a statistically significant association between rs80213143 and both SLE and LN, addressing a critical gap in the genetic landscape of SLE.

*LOC100130476*, located at chromosome 6q23.3, encodes wound and keratinocyte migration-associated lncRNA 2 (WAKMAR2, named by Herter, et al.) and was reported involved in the pathogenesis of gastric cardia adenocarcinoma, malignant progression of esophageal squamous cell carcinoma, and the inflammation of human chronic wounds (Guo et al., 2016a; Guo et al., 2016b; Herter et al., 2019). The methylation-mediated downregulation of *LOC100130476* was detected and might function as a tumor suppressor gene in gastric cardia adenocarcinoma (Guo et al., 2016b). Aberrant hypermethylation of the CpG sites in exon one downregulated the expression of LOC100130476 which was associated with poor esophageal squamous cell carcinoma patient survival (Guo et al., 2016a). Recent advances revealed the protective role of WAKMAR2 in skin wound healing process by restriction of the production of inflammatory chemokines in keratinocytes (Herter et al., 2019). Our findings extend the biological significance of LOC100130476 by linking its genetic variation to autoimmunity, suggesting that alterations in its expression or function may contribute to SLE pathogenesis. Located in LncRNA coding regions, the variant rs13259960, rs205764 and rs547311 were reported associated with increased SLE susceptibility by modulated the expression of SLEAR and linc00513, respectively (Fan et al., 2020; Xue et al., 2018). Advancements in the field indicate that an increasing number of studies suggested genetic variations in the regions of lncRNAs are implicated in susceptibility to SLE. Given the complexity of genetic regulation and the possibility of additional functional variants within this region, further comprehensive studies—including broader sequencing and fine-mapping—are needed to clarify the causal variants and their biological significance.

Subsequently, we explored the potential functional implications of rs80213143. Annotations from RegulomeDB indicate that the rs80213143 locus is situated within a transcription factor binding region. The transition from G to C at this locus affects the binding affinity with various transcription factors, thereby influencing regulatory motifs and leading to reduced binding scores. The C allele, identified as the risk allele at the rs80213143 locus, is associated with a reduced binding capacity to the transcription factors Hand1_1, Hand1_2, RP58, and TAL1_known2. Hand1 and ZBT18 function as transcriptional repressors, with Hand1 notably inhibiting SOX15, and ZBT18 affecting development and cell processes like myogenesis and neuron survival (UniProt Consortium, 2023). Therefore, the decreased binding with these repressive transcription factors by the risk allele C suggested an association with increased gene expression. Following an analysis of the transcriptome across various immune cell subtypes, we observed that the rs80213143 locus did not exert a QTL effect in all immune cell subtypes. Notably, its impact on expression was primarily observed in subsets of monocytes, neutrophils, and T cells. This suggested that rs80213143 might contribute to the pathogenesis of SLE by modulating expression specifically within these immune cell populations. The QTL analysis suggests that the allele C of rs80213143 on RP11-356I2.4 is responsive to sun exposure, implying that this variant could contribute to the pathogenesis of SLE by modulating gene expression levels in an environment-dependent manner.

In the present study, *LOC100130476* expression was significantly upregulated in SLE patients, with the highest levels observed in patients with lupus nephritis. Given the clinical heterogeneity of lupus nephritis, the interpretation of this expression difference requires caution. Renal dysfunction itself may influence gene expression profiles in both peripheral blood and renal tissues. As the present analyses were cross-sectional and included LN patients with varying degrees of renal impairment, causality cannot be inferred from these data alone. The observed upregulation of *LOC100130476* may therefore reflect either a contributing factor to LN pathogenesis or a secondary consequence of renal injury. Future studies focusing on LN patients with preserved renal function, as well as longitudinal analyses correlating gene expression with renal function parameters, will be necessary to clarify the temporal and causal relationship between *LOC100130476* expression and renal involvement.

In the past few years, the correlation between the expression of lncRNA and renal impairment in SLE were revealed. In SLE patients with kidney injury, the expression of LNC-DC was significantly elevated, while the downregulations of linc0949 and lncRNA TUG1 was markedly lower (Wu et al., 2017; Cao et al., 2020). A negative correlation was identified between TUG1 levels and 24-h urinary protein (Cao et al., 2020). Moreover, the level of lncRNA TUG1 was a potential clinical diagnostic tool to distinguish lupus nephritis patients from SLE patients without renal impairment (Cao et al., 2020). Given the number of clinical manifestations examined and the absence of predefined *a priori* hypotheses, the genotype–phenotype association analyses should be regarded as exploratory in nature. After strict correction for multiple testing, the observed associations with renal manifestations would not remain statistically significant and therefore require validation in independent cohorts.

Furthermore, we explored LOC100130476’s role in SLE and LN, revealing its influence on crucial disease pathways. Our analysis revealed significant enrichment in pathways related to T cell receptor, antigen presentation, interferon, and apoptosis, pointing to LOC100130476’s potential in driving SLE pathogenesis. Additionally, LOC100130476 was linked to LN through pathways of leukocyte-mediated immunity, inflammatory responses, pathways related to the extracellular matrix and tissue repair, and the PI3K signaling network, indicating its key role in LN’s pathogenic mechanisms.

Our study suggested that LOC100130476 may have played a promotional role in the pathogenesis of SLE and LN. Given its specific impact on autoimmune conditions, this suggested a targeted role in disease progression. However, the precise functional mechanisms of rs80213143 remain to be elucidated. Future studies could employ functional experiments, such as CRISPR/Cas9 gene editing or *in vitro* cell model validation, to systematically investigate the regulatory effects of rs80213143 on *LOC100130476* expression and downstream immune pathways. These experiments will help clarify the independent contribution of rs80213143 to SLE and LN pathogenesis.

In conclusion, our comprehensive analysis establishes a genetic link between the rs80213143 variant at LOC100130476 and SLE susceptibility, particularly highlighting its impact on renal involvement. These findings enhance our understanding of the molecular mechanisms underlying SLE and lupus nephritis (LN). Moreover, LOC100130476 holds potential as a biomarker for lupus nephritis, which could aid in better diagnosis or monitoring of disease progression. Future studies focused on further elucidating the regulatory mechanisms and functional consequences of this variant will be important for advancing our understanding of its role in SLE and LN.

## Data Availability

The datasets presented in this study can be found in online repositories. The names of the repository/repositories and accession number(s) can be found in the article/Supplementary Material.
