# Supplementary material for: Genetic association of LOC100130476 rs80213143 with susceptibility and renal involvement in systemic lupus erythematosus
Source: Front Genet. 2026 Apr 1;17:1609849. doi: 10.3389/fgene.2026.1609849 (PMC13078731; doi:10.3389/fgene.2026.1609849)
Supplement: Supplementary file 1 [file Table1.docx]

**Genetic Association of LOC100130476 rs80213143 with Susceptibility and Renal Involvement in Systemic Lupus Erythematosus**

Xiao-Xue Zhang^123#^, Jun-Peng You^145#^, Yong-Chun Li^24#^, Hong-De Xu^24^, Xin-Yi Chen^123^, Zhan-Zheng Zhao^123*^, Yuan-Yuan Qi^123*^

**AUTHORS’ INSTITUTION AND AFFILIATION**

1. Department of Nephrology, The First Affiliated Hospital of Zhengzhou University, Zhengzhou Henan, 450052 P. R. China

2. Zhengzhou University, Zhengzhou Henan, 450001 P. R. China

3. Laboratory of Nephrology, The First Affiliated Hospital of Zhengzhou University, Zhengzhou Henan, 450052 P. R. China

4. Ministry of Education of China, Institute of Drug Discovery and Development, Zhengzhou University, 100 Kexue Avenue, Zhengzhou, Henan, 450001, China

5. School of Pharmaceutical Sciences, Zhengzhou University, 100 Ke xue Avenue, Zhengzhou, Henan 450001, China

^#^: These authors contributed equally to this work.

CORRESPONDING AUTHOR

Dr. Yuan-yuan Qi, MD & PhD;

Email: qqyyiillyy@126.com

Nephrology Hospital, the First Affiliated Hospital of Zhengzhou University,

Institute of Nephrology, Zhengzhou University

No.1, Jianshe Road, Erqi District

Dr. Zhan-zheng Zhao, MD & PhD;

Email: zhanzhengzhao@zzu.edu.cn

Nephrology Hospital, the First Affiliated Hospital of Zhengzhou University,

Institute of Nephrology, Zhengzhou University

No.1, Jianshe Road, Erqi District

Zhengzhou 4500052, P.R China

Supplementary table1. Association results of SNPs in *LOC100130476* and SLE susceptibility.

| SNP | Chr. | Pos (hg19) | Minor allele | MAF (Case/Control, %) | P-value | OR (95%CI) |
| --- | --- | --- | --- | --- | --- | --- |
| rs653520 | 6 | 138145552 | G | 16.4/9.8 | 1.51×10^-5^ | 1.80(1.38-2.36) |
| rs657180 | 6 | 138146415 | G | 18.1/11.7 | 6.65×10^-5^ | 1.67(1.30-2.15) |
| rs657597 | 6 | 138146505 | T | 19.4/12.9 | 8.75×10^-5^ | 1.63(1.27-2.08) |
| rs670369 | 6 | 138147048 | C | 17/10.1 | 8.03×10^-6^ | 1.82(1.40-2.37) |
| rs2788288 | 6 | 138149753 | T | 15.9/9.6 | 2.99×10^-5^ | 1.78(1.35-2.33) |
| rs72980748 | 6 | 138150539 | T | 1.4/1.3 | 0.83 | 1.09(0.51-2.32) |
| rs7746779 | 6 | 138154501 | G | 1.4/1.2 | 0.68 | 1.18(0.54-2.56) |
| rs56232106 | 6 | 138156122 | C | 2.8/1.5 | 5.67×10^-2^ | 1.84(0.97-3.48) |
| rs17779870 | 6 | 138156425 | C | 1.3/1.1 | 0.67 | 1.19(0.53-2.67) |
| rs9389536 | 6 | 138157037 | A | 35.4/40.1 | 3.33×10^-2^ | 0.82(0.68-0.98) |
| rs57163170 | 6 | 138157308 | A | 1.4/1.2 | 0.68 | 1.18(0.54-2.56) |
| rs80213143 | 6 | 138157728 | C | 10/4.1 | 2.50×10^-7^ | 2.63(1.80-3.84) |
| rs6918329 | 6 | 138161013 | G | 13.6/7.5 | 1.26×10^-5^ | 1.93(1.43-2.61) |
| rs111883038 | 6 | 138168028 | A | 9.3/3.9 | 1.08×10^-6^ | 2.56(1.73-3.78) |
| rs80126770 | 6 | 138168083 | T | 9.4/3.9 | 7.94×10^-7^ | 2.59(1.75-3.81) |
| ccc-6-138212294-C-A | 6 | 138170601 | A | 11.5/6.8 | 2.72×10^-4^ | 1.79(1.30-2.45) |
| ccc-6-138212299-T-A | 6 | 138170606 | A | 2/2.9 | 0.2 | 0.69(0.39-1.23) |
| rs9494883 | 6 | 138171466 | G | 9.6/4 | 6.51×10^-7^ | 2.58(1.75-3.78) |
| rs600144 | 6 | 138172383 | C | 16.7/17.6 | 0.59 | 0.94(0.74-1.19) |
| rs7753873 | 6 | 138173422 | C | 11.5/6.7 | 1.93×10^-4^ | 1.82(1.32-2.50) |
| rs9376303 | 6 | 138174101 | T | 9.8/7.8 | 0.12 | 1.28(0.94-1.75) |
| rs7767264 | 6 | 138177458 | G | 11.7/6.7 | 1.11×10^-4^ | 1.85(1.35-2.54) |
| ccc-6-138219986-T-C | 6 | 138178293 | C | 10.6/4.6 | 4.05×10^-7^ | 2.48(1.73-3.56) |
| rs11970411 | 6 | 138179161 | C | 10.8/6.1 | 2.78×10^-4^ | 1.85(1.32-2.59) |
| rs73564258 | 6 | 138181604 | A | 0.5/0.6 | 0.77 | 0.84(0.25-2.75) |
| rs7774101 | 6 | 138182492 | G | 11.5/6.4 | 6.94×10^-5^ | 1.91(1.38-2.63) |
| rs9376304 | 6 | 138182995 | C | 33.8/38.8 | 2.24×10^-2^ | 0.81(0.67-0.97) |
| rs9494886 | 6 | 138184330 | G | 11.7/6.7 | 1.11×10^-4^ | 1.85(1.35-2.54) |
| rs59699063 | 6 | 138185881 | T | 11.7/6.5 | 5.32×10^-5^ | 1.92(1.39-2.64) |
| rs61593413 | 6 | 138185932 | A | 11.7/6.5 | 5.32×10^-5^ | 1.92(1.39-2.64) |
| rs59693083 | 6 | 138186532 | G | 11.7/6.5 | 5.32×10^-5^ | 1.92(1.39-2.64) |
